# Radiotherapy combined with immunotherapy successfully treated one case of anaplastic thyroid cancer: A case report

**DOI:** 10.3389/fonc.2023.1125226

**Published:** 2023-05-15

**Authors:** Yurou Xing, Yongsheng Wang, Xin Wu

**Affiliations:** ^1^ Thoracic Oncology Ward, Cancer Center, West China Hospital, Sichuan University, Chengdu, Sichuan, China; ^2^ Head and Neck Oncology Ward, Cancer Center, West China Hospital, Sichuan University, Chengdu, Sichuan, China

**Keywords:** anaplastic thyroid carcinoma, PD-1 inhibitor, radiotherapy, tislelizumab, immunotherapy

## Abstract

**Background:**

Anaplastic thyroid cancer (ATC) is a rare but highly fatal form of thyroid cancer. This highly malignant tumor progresses rapidly and is prone to relapse and metastasis, with a poor prognosis. Novel treatments have improved survival in recent years, but the outcome of treatment is not satisfactory.

**Case presentation:**

We report a case of multiple postoperative recurrences of papillary thyroid carcinoma that later transformed into undifferentiated carcinoma. The patient’s neck mass was huge and the operation was unsuitable. Then, she achieved remarkable tumor shrinkage by tislelizumab immunotherapy combined with radiotherapy.

**Conclusion:**

This case indicates that radiotherapy combined with immunotherapy is a promising treatment for ATC. Such a combined approach warrants further study.

## 1 Introduction

Anaplastic thyroid carcinoma is a highly aggressive and lethal tumor and is associated with a poor prognosis. It is characterized by rapid progression, often accompanied by local infiltration and distant metastasis. ATC is lethal even when detected early and removed, and all undifferentiated thyroid carcinomas are stage IV tumors. The main treatments are surgery, radiotherapy and systemic therapy (1). In recent years, new treatment methods have improved the prognosis of ATC and brought hope to the treatment of ATC. In this case, we present a patient with ATC who had sustained tumor remission after receiving tislelizumab (a monoclonal antibody against the PD-1 receptor) and radiotherapy to the neck.

## 2 Case presentation

**Figure 1:**
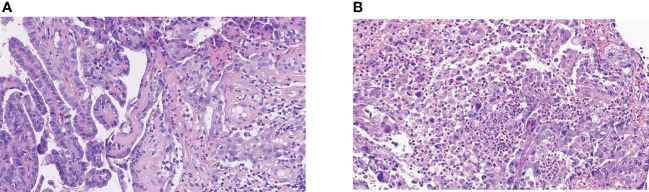
The pathology of the patient. **(A)** Well-differentiated thyroid papillary carcinoma at initial. **(B)** Anaplastic change from papillary carcinoma of the right neck lymph node. The anaplastic carcinoma is composed of tumor cells in a nested pattern with markedly enlarged pleomorphic and hyperchromatic nuclei and an eosinophilic cytoplasm. Typical papillary carcinoma-like nuclear features (ground glass and grooving) are absent. The stroma is richly infiltrated with inflammatory cells.

**Figure 2:**
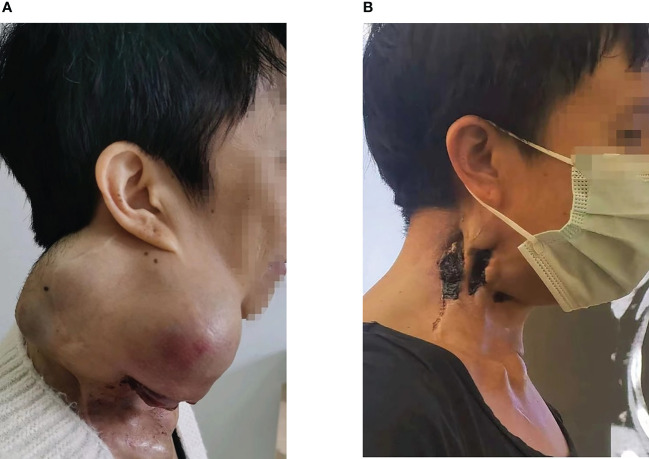
**(A)** The right neck mass, about 26cm*18cm*15cm in size, locally ruptured and bled in June 2021. **(B)** After radiotherapy and immunotherapy, the patient’s neck mass shrank, as shown above in July 2022.

**Figure 3:**
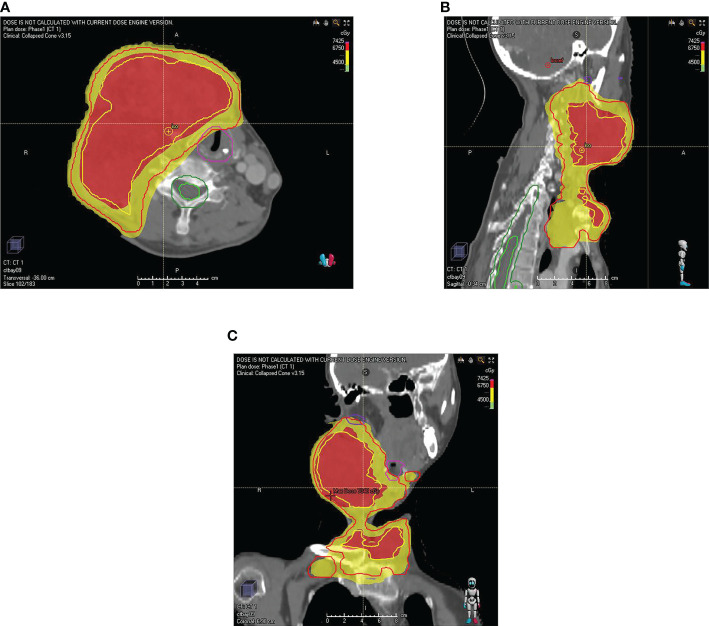
The map of the patient’s radiotherapy target volume. **(A)** transverse position **(B)** sagittal position **(C)** coronal position. The dose was 6750 cGy in the red part (GTVboost) and 4500 cGy (GTV) in the yellow part.

**Figure 4:**
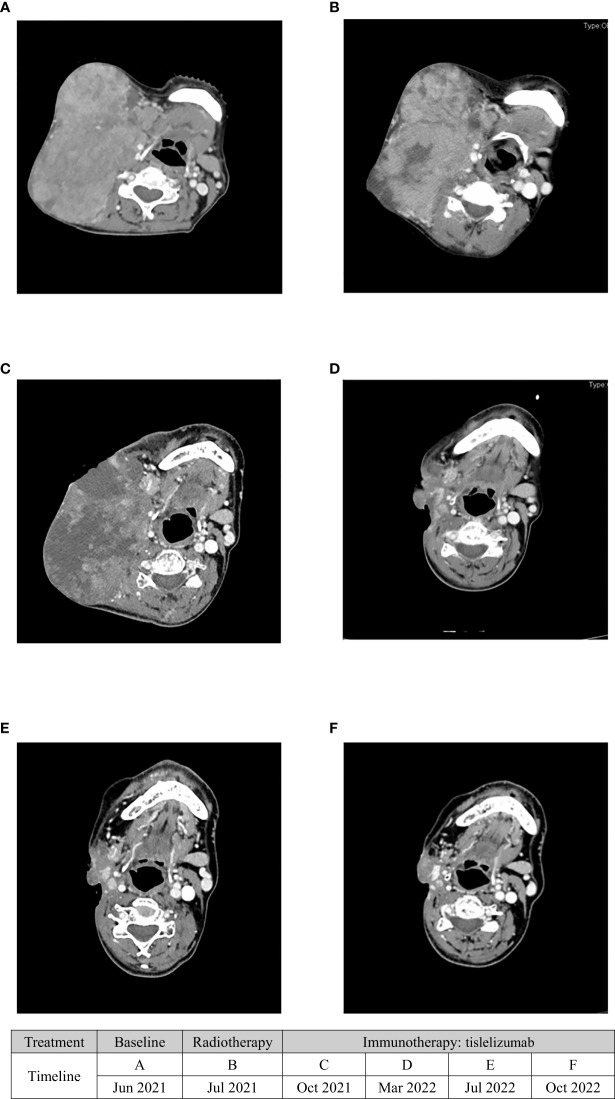
The serial CT images of the patient before and after treatment with radiotherapy and tislelizumab immunotherapy. **(A)** CT image of right neck lymph node recurrence in June 2021(baseline). **(B)** CT image of the right neck lymph node in July 2021 after radiotherapy. **(C)** CT image of the right neck lymph node in October after tislelizumab immunotherapy. **(D)** CT image of the right neck lymph node in March 2022. It showed the tumor shrank. **(E)** CT image of the right neck lymph node in July 2022. **(F)** CT image of the right neck lymph node in October 2022.

A 47-year-old woman was diagnosed with anaplastic thyroid carcinoma. She came to our hospital with a huge mass in her neck. She had no remarkable past medical history or family history. In 2002, the patient was found to have a thyroid mass, and then underwent a total thyroidectomy and central neck lymph node dissection. Postoperative pathology revealed that the right thyroid mass was classic papillary carcinoma (**Figure 1A**). Three lymph node metastases were identified and she did not receive postoperative iodine 131 therapy after the operation. The patient’s review indicated tumor recurrence in 2008 and she received right neck lateral lymph node dissection. A total of 4/11 lymph nodes dissected during surgery were diagnosed with metastatic carcinoma. The patient refused iodine-131 therapy due to financial problems. The patient was reexamined regularly after surgery, and no signs of tumor recurrence were found. Unfortunately, the reexamination of the patient in 2013 showed tumor recurrence and she was given four sessions of radioiodine therapy with iodine-131. The dose ranged from 150 to 200 mci per session. At the last treatment, the tumor did not uptake iodine, suggesting that the tumor did not respond to iodine-131 therapy. The patient went to a hospital in Shanghai for consultation on treatment in 2016, and right lateral cervical lymph node dissection was performed. The patient was treated with oral Chinese medicine after surgery. In 2019, the patient again had a mass in the right neck, which gradually increased rapidly in size. The patient was not suitable for surgery because the tumor was encased in blood vessels, of severe trauma and higher risks. Pathological results of core needle biopsy confirmed that the right neck mass was undifferentiated thyroid carcinoma, CK(+),TTF-1(focal +),TG(focal +),PAX8(+),CD117(-),CD5(-),CgA(-),Syn(-),Calcitonin(-)PTH(-),P63(-), Ki-67 60%(**Figure 1B**). The results of genetic testing showed that BRAF V600E was negative and TERT gene was positive. No targetable gene mutation was found in the patient and the immune index PDL1 was positive (PDL1 22C3, CPS=10). Because the mass continued to bleed, she did not receive antineoplastic therapy. The patient presented to our hospital with a large, locally ruptured and bleeding thyroid mass in June 2021.The bleeding volume of the mass was approximately 100 ml per day, resulting in severe anemia (**Figure 2A**). Routine blood tests showed hemoglobin 48 g/L. Physical examination revealed a hard mass on the right neck, approximately 26 cm*18 cm*15 cm in size, with local skin ulceration and bloody secretion. The patient had anemic appearance and pale complexion, lips, eyelids and conjunctiva. The results of general examination showed that the patient had multiple metastatic tumors in both lungs. After comprehensive evaluation by multiple disciplines (including thyroid surgery, oncology, radiotherapy, nuclear medicine, etc.) in our hospital, a comprehensive treatment strategy was developed. The surgeon considered that the patient had undergone several previous neck surgeries and that complete resection of the tumor would be difficult with reoperation. Radiotherapy was recommended for the patient. The patient received radiation therapy with a prescription of 4500 cGy in 15 fractions to the right neck mass and 6750 cGy in 15 fractions to the tumor boost (the target area of radiotherapy shrunk the edge of the mass by 1 cm and avoided the large blood vessels and nerves and other tissues) using a simultaneous integrated boost technique, IMRT IGRT (**Figure 3**). Subsequently, tislelizumab immunotherapy was recommended as a treatment choice. The patient has been treated with tislelizumab at a dose of 200 mg every three weeks from July 2021. Tislelizumab immunotherapy was well tolerated and almost no adverse reactions were observed in the patient. Her neck mass was gradually reduced in size after radiotherapy and tislelizumab immunotherapy. (**Figure 2B**). Serial CT images of the patient are shown in (**Figure 4**). The lung metastatic lesions were smaller than before, and the efficacy evaluation was partial response. To date, no tumor recurrence has been observed. The blood test revealed that the hemoglobin level gradually increased (97 g/L). At present, the patient can move the neck freely and no longer bleeding. The patient’s physical discomfort such as fatigue and neck pain disappeared. The patient was highly motivated to receive maintenance treatment and follow-up due to the excellent curative effect. She felt energetic and was able to go to work as normally.

## 3 Discussion

Anaplastic thyroid carcinoma is a very rare tumor that accounts for 1–2% of all thyroid cancers. The incidence of ATC is extremely low (2). ATC is more common in elderly individuals and in females, and most of them are diagnosed at an advanced stage due to the extreme aggressiveness of the tumor (3). The most common symptom of ATC is a rapidly enlarging neck mass, accompanied by some symptoms of local compression, including hoarseness, dysphagia, and dyspnea (4). Distant metastasis is common in ATC, especially in the lung, bone, and brain. Anaplastic thyroid carcinoma has an extremely poor prognosis, the median survival is 5 months, and 1-year survival rate is 20% (5).

ATC cells are derived from thyroid follicular cells but have lost the function of normal cells. ATC tumorigenesis is considered as a multi-step dedifferentiation process (6). Some cases were found to coexist with ATC and DTC, suggesting that some ATC cells may develop from well-differentiated thyroid cancers (7). This histological transformation leading to increased malignancy may be related to thyroidectomy or I131 or thiouracil treatment (8).

ATC is often manifests as a rapidly growing neck mass and early diagnosis of ATC is particularly important. This patient underwent core needle biopsy for the diagnosis of anaplastic thyroid carcinoma. Surgical routine biopsy is traumatic and risky, which is not the best choice. In the past, fine needle aspiration biopsy was mainly used, but it could not provide tissue information on some highly malignant tumors. A recent study showed that core needle biopsy had a higher diagnostic rate and could identify specific tissue types and molecular analysis to guide clinical medication. Its advantages include being safe and well tolerated, and the incidence of hematoma, edema, infection and other complications is low. This strongly supports the use of CNB in the diagnosis of giant masses in the thyroid gland (9, 10).

Treatment options available for ATC include surgery, radiotherapy, chemotherapy, targeted therapy and immunotherapy. Factors associated with improved survival were absence of lymph node involvement, absence of metastasis, tumor size ≤6 cm, surgery, radiotherapy, and chemotherapy (11). A meta-analysis demonstrated that surgery improved OS in ATC patients without distant metastases (12). For patients with localized ATC, surgical treatment is an appropriate choice. Given the rapid progression of ATC, radiotherapy and systemic therapy are recommended as soon as possible after surgery (6). Due to local invasion and distant metastasis, ATC is often difficult to cure by surgery. In this case, due to multiple neck operations, it was difficult to completely remove the tumor by reoperation, and the postoperative prognosis was poor. In the end, surgery was not performed.

An analysis of data from the SEER database showed that chemoradiotherapy improved OS in patients with ATC. For ATC patients without targeted gene mutations, chemoradiotherapy is a recommended treatment (13). The most commonly used chemotherapy drugs include paclitaxel, doxorubicin, or combined therapies (6). In view of the patient’s poor general condition, severe anemia, and poor tolerance to chemotherapy, chemotherapy was not performed.

Recent studies have demonstrated that several genetic alterations and activation of signaling pathways are associated with the occurrence of ATC, such as BRAF, TP53, TERT mutation and activation of the PI3K-AKT signaling pathway (14–16). BRAF mutations are seen in approximately 45%-74% of ATCs (17, 18). The combination of the BRAF inhibitor dabrafenib and the MEK inhibitor trametinib demonstrated robust clinical activity in the treatment of undifferentiated thyroid cancer with BRAF mutations, resulting in a confirmed overall response rate of 69% (19, 20). One study revealed that the 1-year overall survival rate reached 94% in ATC patients with BRAF mutation who received neoadjuvant BRAF-directed therapy followed by surgery (21). These encouraging results hold promise for the personalized treatment of ATC. TERT gene mutation was also found to be a common mutation in ATC patients. However, there is still no effective targeted drug for TERT mutation (7). In addition, everolimus has been reported to exert antitumor activity in ATC by blocking the PI3K/AKT/mTOR pathway in some phase II clinical studies (22). In this case, the BRAF gene test was negative, and there was no indication to use dabrafenib and trametinib targeted therapy.

Vascular endothelial growth factor (VEGF) can induce the formation of new blood vessels, which is related to the distant metastasis of tumors (23). VEGF is commonly found in highly malignant ATC cells. A multitargeted inhibitor, lenvatinib, showed promising activity and a manageable toxicity profile in anaplastic thyroid cancer in a phase 2 trial. The median PFS of ATC patients was 7.4 months, and the median OS was 10.6 months (24). Other oral multitarget TKI drugs, such as sorafenib, have also been used in the treatment of ATC (25). The patient was not treated with antiangiogenic therapy because of severe bleeding from the neck mass.

The binding of the programmed death-1 (PD-1) receptor and its ligand programmed death-ligand 1/2 (PD-L1/L2) suppresses the immune response of cytotoxic T-cells. Drugs targeting this mechanism have shown clinical activity in a variety of tumors. PDL1 expression is increased of in ATC cells (22.2%-28.6%) (26, 27). Targeted PD-L1 therapy is a promising treatment for patients with advanced ATC (28). Spartalizumab achieved a higher response rate in PD-L1 positive patients (ORR=29%) than in PD-L1 negative patients. The 1-year survival rate was 52.1% in PD-L1 positive ATC patients (29). In addition, a clinical study showed that pembrolizumab combined with lenvatinib have also achieved good therapeutic effects in ATC. A total of 4/6 patients had a complete response and median progression-free survival was 16.5 months for ATCs (28). There has been a case report of substantial regression of tumor nodules in ATC patients treated with nivolumab and vemurafenib (30). Another case report showed that sintilimab and anlotinib have shown durable responses in ATC (31). The use of tislelizumab led to sustained tumor remission in this case. Tislelizumab is a human IgG4 monoclonal antibody. It binds to the PD1 receptor on the surface of immune cells, thereby blocking the binding of PD-L1 on the surface of tumor cells and PD1. Moreover, tislelizumab has a higher affinity than pembrolizumab and nivolumab, resulting in more effective anti-tumor effect. Macrophages express Fc receptors, and the combination of anti-PD-1 antibodies (such as nivolumab and pembrolizumab) and Fc receptors results in the connection of T cells expressing PD-1 with macrophages expressing Fc receptors. The efficacy of anti-PD-1 antibody may be weakened by the phagocytosis of T cells by macrophages. This phagocytosis is eliminated due to the low affinity of tislelizumab for FC receptors (32). Patients can obtain a high remission rate.

In recent years, radiotherapy technology has been gradually improved. The application of IMRT makes the radiotherapy range more precise and has fewer adverse reactions. Radiotherapy has emerged as an alternative treatment for unresectable ATC. Previous series of studies have suggested that higher doses of radiation can enhance the disease response rate (11, 33). Preclinical studies have demonstrated that hypofractionated radiotherapy significantly slows tumor growth and metastasis development. Hypofractionated RT has advantages over conventionally fractionated RT in improving local control, metastatic spread, and prolonging survival (34). A retrospective study demonstrated that hypofractionated radiotherapy reduces local recurrence mortality in patients with ATC. Some investigators have found a trend toward better OS benefits with hyperfractionated radiotherapy. However, it did not improve the OS of patients (35). Kebebew and colleagues have reported that surgical resection and postoperative high-dose radiotherapy are associated with lower cause-specific mortality in ATC (36). A multivariate analysis including 95 ATC patients confirmed that multimodal treatment with surgery and chemoradiotherapy had higher disease-specific survival (37). Similarly, Rao and colleagues assessed a survival of 22.1 months in patients treated with multimodal therapy, as compared with 11.9 months in the general population (4). This proves that radiotherapy is valuable in ATC patients.

Radiotherapy and immunotherapy have synergistic antitumor effects. It has been demonstrated that PDL1 expression in tumor cells increases after radiotherapy, and immunotherapy can overcome this radiation resistance (38). Radiotherapy can promote the release of inflammatory factors and immune stimulators, making the tumor more immunogenic and enhancing the effect of immunotherapy. This effect has been verified in models such as lung cancer (39). Higher doses of radiotherapy can induce cell necrosis and senescence and are considered to be more pro-inflammatory. In addition, high doses of radiotherapy promote the release of cytokines to induce the activation of cytotoxic T cells and initiate the cellular immune response. High-dose radiotherapy combined with immunotherapy is a synergistic mode with good protection of surrounding tissues and fewer adverse reactions. Under this mode, the tumor control rate is high, and patients enjoy a higher quality of life (40, 41). In the past, some cases have reported sustained remission of ATC after radiotherapy combined with pembrolizumab (42). In contrast to conventional fractionated RT, the induction of cell necrosis by hypofractionated radiotherapy is more conducive to the initiation of the cellular immune response (40). For this patient, field-in-field radiotherapy was adopted. On the one hand, a high dose can be delivered to part of the tumor volume safely, which can better kill tumor cells in the hypoxic area of the mass and eliminate radiotherapy resistance. On the other hand, lower doses provided full coverage of the tumor area, stimulating immune activity and increasing the efficacy of immunotherapy. The optimal combination of radiotherapy and immunotherapy is also being actively explored. A phase II trial of pembrolizumab immunotherapy after IMRT in ATC is also currently being recruited (NCT05059470).

## 4 Conclusion

We have reported an ATC case treated with a combination of RT and tislelizumab. The patient’s large neck mass was reduced after treatment, and this effect is quite uncommon in clinical practice. This combination is well tolerated in ATC. Our results indicate that radiotherapy combined with immunotherapy is a promising treatment for ATC. Such a combined approach warrants further study.

## Data availability statement

The original contributions presented in the study are included in the article/supplementary material. Further inquiries can be directed to the corresponding author.

## Ethics statement

Written informed consent was obtained from the patient for the publication of any potentially identifiable images or data included in this article.

## Author contributions

XW designed the study and edited the final manuscript. YX collected the clinical data and wrote the manuscript. YW revised the article. YX and YW contributed equally to this manuscript. All authors contributed to the article and approved the submitted version.
